# Computational Design of Binding Proteins to EGFR Domain II

**DOI:** 10.1371/journal.pone.0092513

**Published:** 2014-04-07

**Authors:** Yoon Sup Choi, Soomin Yoon, Kyung-Lock Kim, Jiho Yoo, Parkyong Song, Minsoo Kim, Young-Eun Shin, Won Jun Yang, Jung-eun Noh, Hyun-soo Cho, Sanguk Kim, Junho Chung, Sung Ho Ryu

**Affiliations:** 1 Cancer Research Institute, Seoul National University School of Medicine, Seoul, Republic of Korea; 2 School of Interdisciplinary Bioscience and Bioengineering (I-Bio), Pohang University of Science and Technology (POSTECH), Pohang, Republic of Korea; 3 Department of Biochemistry and Molecular Biology, Seoul National University School of Medicine, Seoul, Republic of Korea; 4 Division of Integrative Bioscience and Biotechnology, Pohang University of Science and Technology (POSTECH), Pohang, Republic of Korea; 5 Department of Systems Biology, College of Life Science and Biotechnology, Yonsei University, Seoul, Republic of Korea; 6 Scripps Korea Antibody Institute, Chuncheon, Republic of Korea; 7 Division of IT Convergence Engineering, Pohang University of Science and Technology (POSTECH), Pohang, Republic of Korea; 8 KT Institute of Convergence Technology, Seocho-gu, Seoul, Korea; Monash University, Australia

## Abstract

We developed a process to produce novel interactions between two previously unrelated proteins. This process selects protein scaffolds and designs protein interfaces that bind to a surface patch of interest on a target protein. Scaffolds with shapes complementary to the target surface patch were screened using an exhaustive computational search of the human proteome and optimized by directed evolution using phage display. This method was applied to successfully design scaffolds that bind to epidermal growth factor receptor (EGFR) domain II, the interface of EGFR dimerization, with high reactivity toward the target surface patch of EGFR domain II. One potential application of these tailor-made protein interactions is the development of therapeutic agents against specific protein targets.

## Introduction

Protein interaction networks evolve over time to create new protein interactions, which results in the dynamic rewiring of links among pre-existing nodes [1]–[3]. The current approaches to develop novel protein interactions are based on gene duplication and gene modification [1]. Gene duplication results in the addition of both a network node (i.e., protein) and links (i.e., interactions) to the protein interaction network [2], [4], [5]. Gene modification, which usually involves point mutations, results in the addition of links to the network [1], [6].

Recent attempts to develop artificial binding proteins, which are based on a single protein framework, have been successful [7]–[9]. In these studies, a large number of random mutations have been introduced into predefined structural regions of protein frameworks, such as fibronectins [10]–[13], lipocalins [14]–[16], and the ankyrin repeat protein motif [17]–[19]. However, although the scaffolds constructed in these studies have shown affinity to various targets, the selection of different protein frameworks specific to a predetermined target surface patch has not been successful except in a recent study that developed protein binders for influenza hemagglutinin [20].

**Figure 1:**
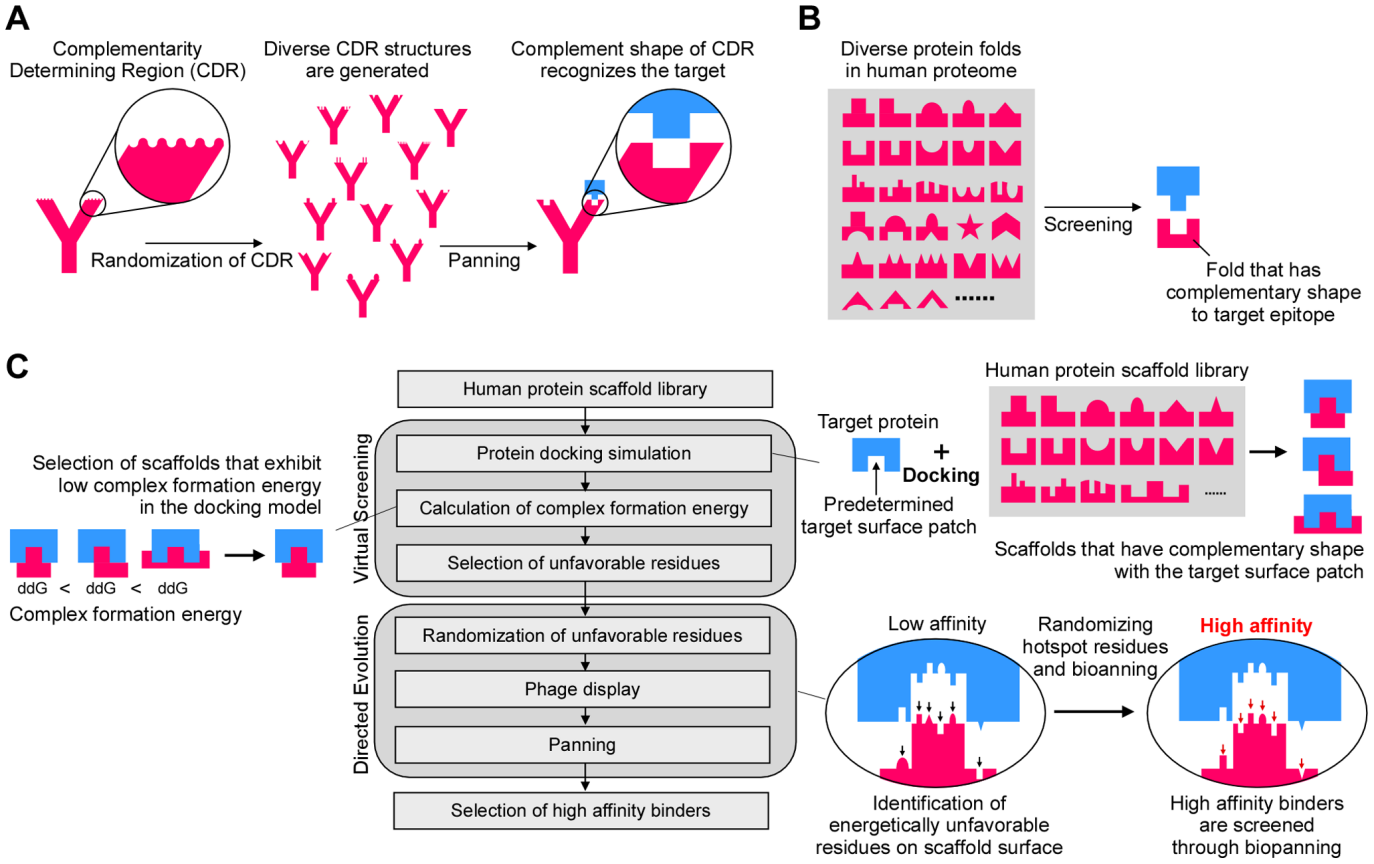
Design scheme of target-specific scaffolds. (A) Synthetic antibodies can achieve extremely diverse structures through sequence randomization of the complementarity determining region (CDR). Among diverse structures, only antibodies with complementary shapes are able to recognize and bind to a particular epitope. (B) By imitating synthetic antibody generation, we devised a strategy to select target-specific scaffolds from the human proteome with shapes that are complementary to the target surface patch. (C) The flow chart shows a two-step strategy to obtain target-specific scaffolds (middle). In the first step, a virtual screening of a human protein scaffold library is conducted to determine a framework specific to the surface patch of interest. Target specific-scaffolds with shapes complementary to the surface patch of interest are selected from the scaffold library through protein docking simulations (upper right). The scaffold–target docking structures with the most favorable complex formation energies are further evaluated (left). In the second step, the scaffold interface in the selected scaffold–target model is optimized by sequence randomization and phage display using directed evolution (lower right).

To mimic the evolutionary process by which protein networks evolve, we adopted the basic mechanism by which antibodies are produced against antigens. When animals are exposed to an antigen, B cells that express a low-affinity surface immunoglobulin are selected. During rapid B-cell proliferation, random mutations are introduced into the immunoglobulin sequences, and clones that express antibodies with high affinities are preferentially selected. To bind a specific antigen with high specificity and affinity, antibodies form a complementary shape to the target surface patch of the antigen using complementarity determining regions (CDRs). The amino acids in CDRs can produce extremely diverse structures, each of which forms the complement shape that recognizes a specific epitope (Figure 1A).

We have developed a strategy using protein docking simulation that imitates this process of antibody generation to select human protein scaffolds with complementary shapes (Figure 1B). This procedure designs novel protein interactions by selecting human protein scaffolds with shapes that complement a predetermined surface patch on a target protein (Figure 1C). In this procedure, key residues are optimized by using an amino acid residue randomization and phage display. The successful implementation of this strategy enables the reproduction of novel protein–protein interactions in the laboratory setting.

**Figure 2:**
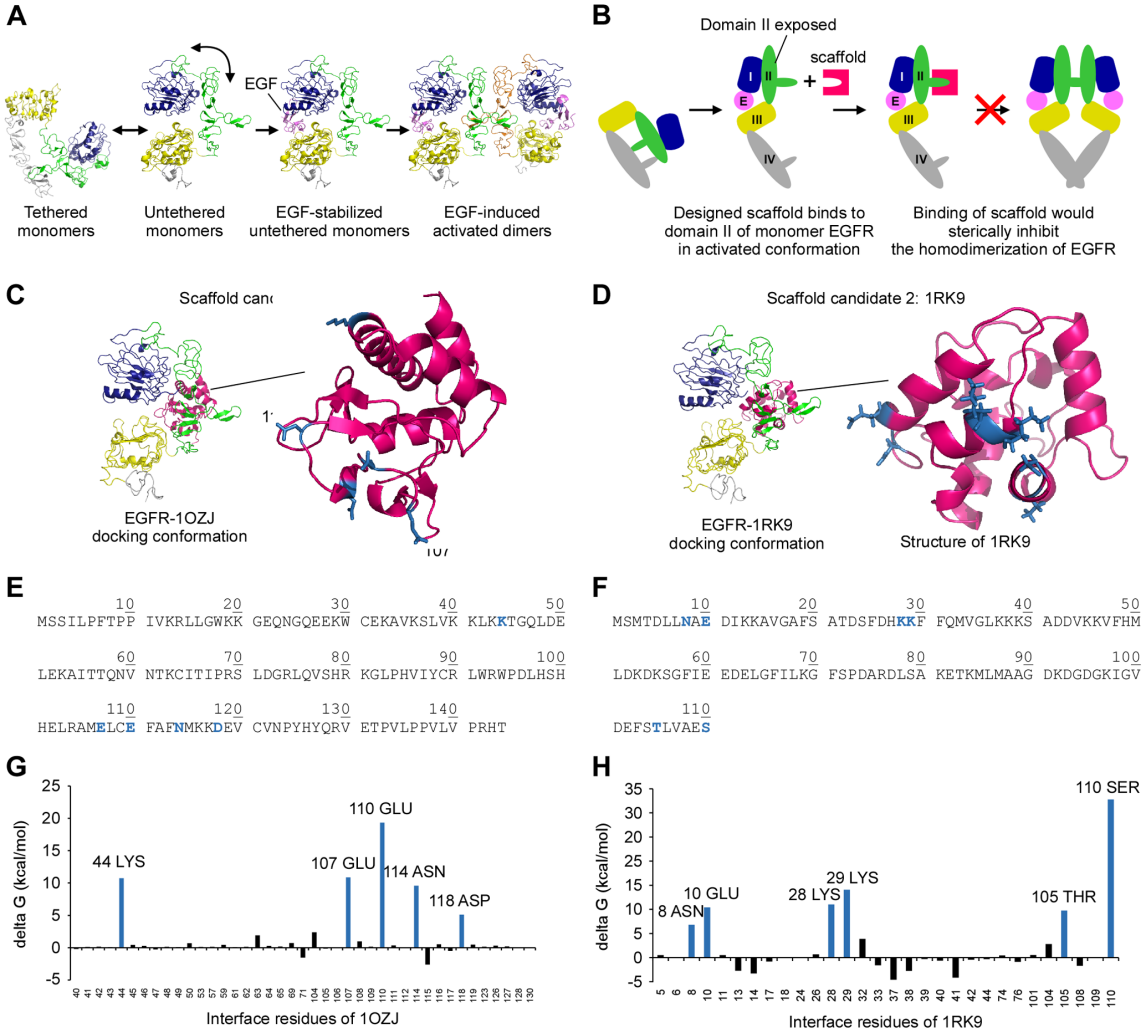
Scaffolds 1OZJ and 1RK9 have shapes complementary to EGFR domain II. (A) The inactivated EGFR monomer exists in equilibrium between the tethered and untethered conformations. The binding of EGF (pink) stabilizes the untethered monomers, which exposes the dimerization arm of domain II (green) and activates EGFR to form homodimers at the domain II dimeric interface (domain I, blue; domain II, green; domain III, yellow; domain IV, gray). For clarity, domain II on the right-hand EGFR in the homodimer is shown in orange. (B) EGFR activation can be blocked by binding domain II, which is exposed in the untethered conformation, with the designed scaffold (magenta), which sterically interferes with EGFR dimerization. (C) The docking conformation of 1OZJ–EGFR, which was selected from the virtual screening procedure (left), and the enlarged 1OZJ structure (magenta). (D) The docking conformation of 1RK9–EGFR, which was selected from the virtual screening procedure (left), and the enlarged 1RK9 structure (magenta). Energetically unfavorable residues that were selected for optimization are depicted in blue. (E–F) Amino acid sequences for 1OZJ and 1RK9, respectively. The blue residues represent amino acid residues that were energetically unfavorable for EGFR complex formation. (G–H) The complex formation energy of the scaffold interface residues upon the formation of the 1OZJ–EGFR and 1RK9–EGFR docking complexes, respectively. The blue bars depict the energetically unfavorable residues that were selected for optimization. The horizontal axis shows each amino acid in the scaffold interface, and the vertical axis represents the energy contribution (delta G) to the complex formation.

We have applied this method to the development of proteins that bind epidermal growth factor receptor (EGFR) domain II. EGFR, which is also known as ErbB1 and HER1, is one of the most extensively studied proteins, and plays key roles in many cancers, including colorectal and lung cancer [21]–[24]. EGFR undergoes a dramatic conformational change when activated to form homodimers or heterodimers with other receptors in the EGFR family [25], [26]. In the absence of the EGF ligand, monomeric EGFR exists in a conformational equilibrium of tethered and untethered states (Figure 2A) [27]. The binding of EGF stabilizes EGFR in the untethered conformation and exposes domain II, which is otherwise occluded by intramolecular interactions, to form the homodimeric interface (Figure 2A) [25]. Binding EGFR domain II when EGFR is in the transition state inhibits EGFR activation. For example, monoclonal antibody 806 primarily binds domain II of an EGFR mutant [28]–[30].

Here, we generated six different protein binders from two different frameworks that have been computationally screened from the human proteome. We show that these binders have a high affinity for EGFR and EGFR fragments that harbor domain II. Moreover, we show that treatment with exogenous EGF further increases the reactivity of these binders to EGFR, which might suggest that these protein binders react with EGFR in its transition state. This ability to develop artificial protein binding pairs provides us with an experimental tool to create new protein interactions in a laboratory setting. Our results suggest that the non-interface surface of a protein can be changed into a *de novo* protein interface.

## Results

### Computational design scheme

We hypothesized that new protein interactions can be created in nature when two proteins share surface patches with complementary structures, and critical hotspot residues on these complementary surfaces are changed to more energetically favorable residues. We therefore devised a two-step strategy to create novel protein-protein interactions in a laboratory setting (Figure 1C). The first step involved virtual screening of protein scaffolds to find those with structural complementarity to the target surface patch and with favorable complex formation energy. In the second step, we improved the reactivity between the scaffold and target proteins using a directed evolution approach (second part of flowchart in Figure 1C). In this two-step strategy, we used the computational components to assist the directed evolution by selecting protein frameworks and narrowing down the potential interfacial residues based on the protein structure and the calculation of the binding energy.

### Virtual screening of scaffolds complementary to EGFR domain II

We first constructed a massive *in silico* library of human protein scaffolds covering virtually all known human protein folds (see the Materials and Methods). The resulting library consisted of 716 semi-redundant human protein structures (Dataset S1 in File S1), which we used to screen for scaffolds that bind the target surface patch.

Subsequently, we conducted a protein docking simulation using the untethered monomer structure of EGFR, specifically chain A of the crystal structure of EGFR (Protein Data Bank [PDB] ID: 1IVO), to select scaffolds from the scaffold library with a surface shape complementary to EGFR domain II. All 716 scaffolds were evaluated using PatchDock [31], [32]. For this rigid-body docking simulation, we assumed that the proteins are not undergoing large conformational changes upon binding.

Each docking attempt generated multiple binding modes, which were scored and ranked by PatchDock based on the complementarity of the protein–protein interface of the docking structures. We found that 708 out of 716 scaffolds (98.9%) generated more than 10 docking models. PatchDock determines the number of binding modes based on the number of complementary protein surface patches between the two input proteins. To select docking conformations with good shape complementarity, we chose the top 10 models for each scaffold for a total of 7,080 docking conformations. We examined the interface residues of each complex to select models in which EGFR domain II was involved in the complex formation. For this step, we chose docking structures in which at least 10 of the 28 residues of EGFR domain II were buried in the interface, which resulted in a total of 1,680 docking complexes selected from 340 scaffolds.

### Selection of scaffolds with favorable binding energy and identification of unfavorable key interface residues

From the 1,680 selected docking complexes, the two scaffolds with the most favorable binding energies (Figure 1C, left) were selected: the B chain of 1OZJ (Smad3 MH1 domain) [33] (Figure 2C) and the A chain of 1RK9 (human alpha-parvalbumin) [34] (Figure 2D). The binding energies were evaluated with the EGAD program, which was developed to calculate the residue interaction energies [35]. The amino acid sequences of 1OZJ and 1RK9 are shown in Figure 2E and 2F, respectively. The energy contributions of each interface residue of 1OZJ (Figure 2G) and 1RK9 (Figure 2H) were assessed by EGAD to select residues unfavorable to complex formation. Five unfavorable interface residues from 1OZJ and six unfavorable interface residues from 1RK9 were selected for randomization (shown in dark blue on the scaffold structures in Figure 2C and 2D, respectively, and in the amino acid sequences in Figure 2E and 2F, respectively).

### Interface refinement through directed evolution using sequence randomization and phage display

We optimized unfavorable interaction residues on the surface of scaffolds 1OZJ and 1RK9 through residue-specific sequence randomization (Figure 1C). High-complexity phage display libraries for 1OZJ and 1RK9 were constructed, and the five critical residues of 1OZJ and six residues of 1RK9 were fully randomized to cover all possible nucleotide combinations. The final size of the 1OZJ library (4.0×10^8^ combinations) was sufficient to cover all possible codon combinations. The 1RK9 library, which theoretically needed 1.1×10^9^ combinations to cover all possible combinations, contained 8.7×10^8^ combinations.

**Figure 3:**
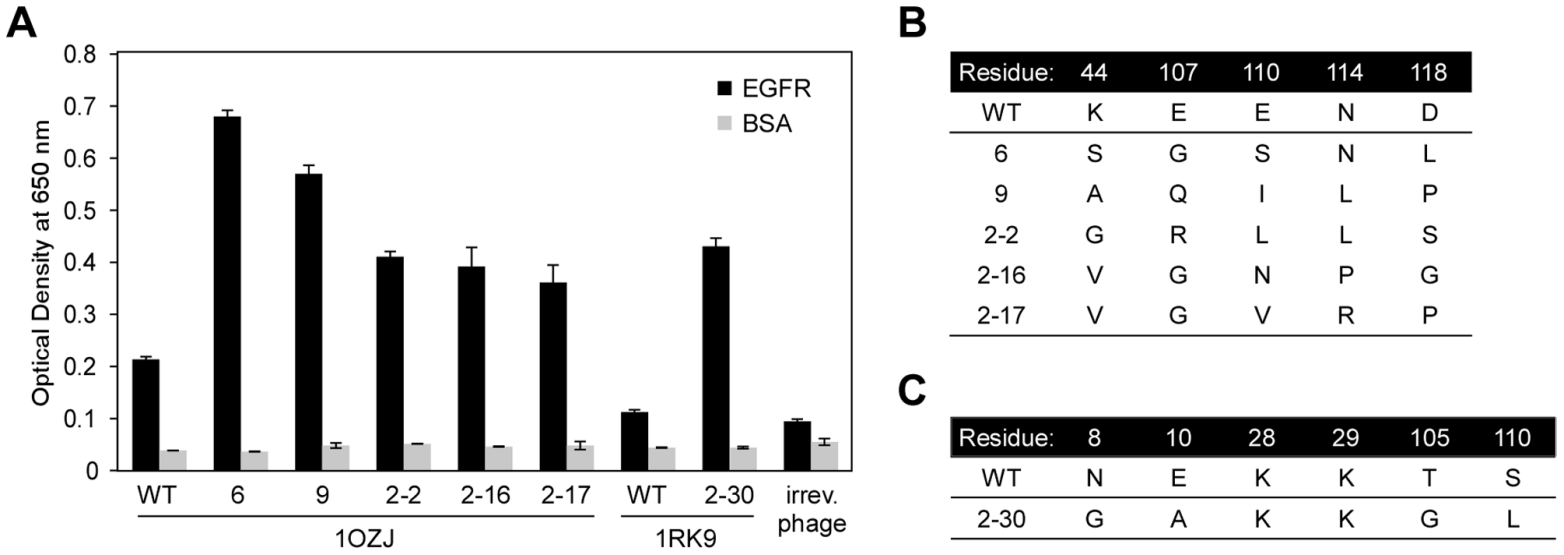
Strong binders were enriched by directed evolution of the 1OZJ and 1RK9 scaffolds. (A) The reactivity of the protein binders (wild-type and mutant clones) against EGFR (black) and BSA (gray) as assessed by phage-ELISA. Error bars represent the standard deviation of triplicate measures. (B) The sequence of the mutant clones generated from 1OZJ. (C) The sequence of the mutant clone generated from 1RK9.

The libraries were subjected to five rounds of biopanning against EGFR-crosslinked magnetic beads to enrich for binders. Clones from the output titer plate of the final round of biopanning were subjected to phage-ELISA (Figure 3A). Five clones of 1OZJ and one clone of 1RK9 were selected for further analysis because their reactivity was significantly higher than that of wild-type 1OZJ and 1RK9 phages. Altered amino acid sequences of the selected 1OZJ and 1RK9 mutant clones are shown in Figure 3B and 3C, respectively. No consensus sequences were found among the high-reactivity binders of 1OZJ. However, we found that bulky amino acids (e.g., Glu, Lys, Asp) were replaced by smaller amino acids (e.g., Ala, Val, Ser).

### Binders showed reactivity against EGFR fragments containing domain II

**Figure 4:**
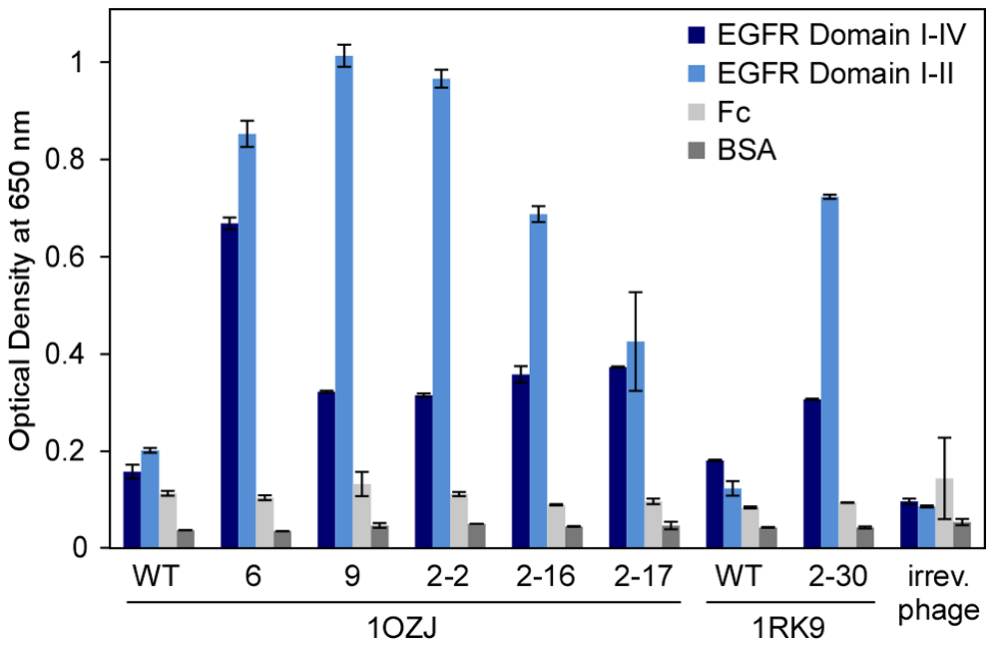
Binders generated from the 1OZJ and 1RK9 scaffolds bind to EGFR fragments I–IV and I–II. The reactivity of the protein binders (wild-type and mutant clones) against EGFR domain I–IV (dark blue) and EGFR domain I–II (light blue) as assessed by phage-ELISA. Error bars represent the standard deviation of triplicate phage-ELISA experiments.

We confirmed that the binders obtained from our directed evolution approach were specific to the EGFR domain II by using fragments of the protein. As shown in Figure 4, all six binders showed reactivity to an EGFR fragment containing domains I–IV (i.e., extracellular region of EGFR, dark blue) and an EGFR fragment containing domains I–II (light blue). Human IgG_1_ Fc fragments and bovine serum albumin (BSA) were used as controls because the EGFR fragments were expressed and purified as Fc fusion proteins. We were unable to express a sufficient amount of an EGFR fragment containing domain II only.

**Figure 5:**
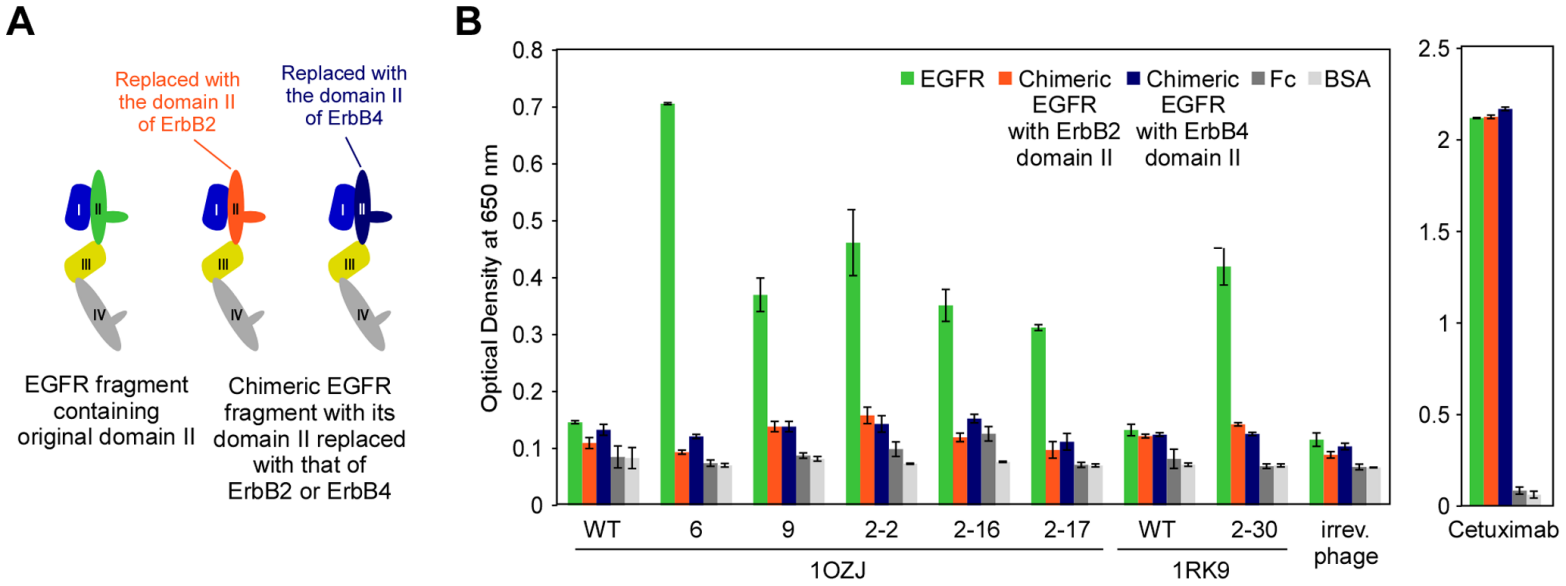
Reactivity of the binders to EGFR depended on the presence of EGFR domain II. (A) Chimeric EGFR fragments were generated with the EGFR domain II replaced with domain II of either ErbB2 (orange) or ErbB4 (dark blue). (B) The reactivity of the protein binders to the EGFR fragments containing EGFR domain II (green), the chimeric EGFR fragments with ErbB2 domain II (orange), and the chimeric EGFR fragments with ErbB4 domain II (dark blue), as assessed by phage-ELISA. Error bars represent the standard deviation of triplicate phage-ELISA experiments.

To further test the specificity of selected binders, we generated chimeric EGFR fragments with the EGFR domain II replaced with domain II of ErbB2 or ErbB4 (Figure 5A). While all six binders showed reactivity to the fragments containing EGFR domain II (green bars), they did not show reactivity to the chimeric fragments containing ErbB2 domain II (orange bars) or ErbB4 domain II (dark blue bars, Figure 5B). Cetuximab, which binds to EGFR domain III [36], retained its reactivity to the chimeric EGFR fragments. Thus, the reactivity of the binders to EGFR depended on the presence of EGFR domain II.

**Figure 6:**
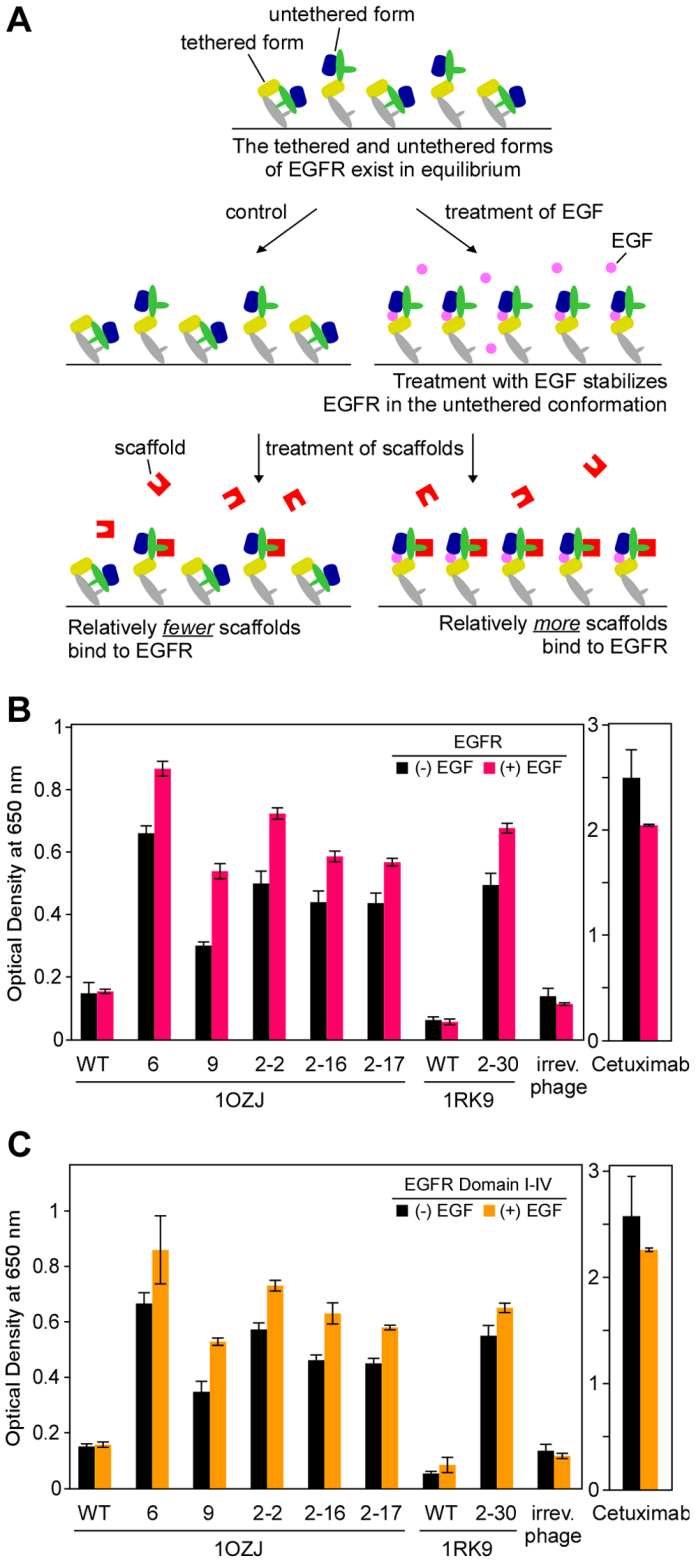
Binders have a higher reactivity to EGFR in the presence of ligand EGF. (A) Ligand EGF stabilizes the untethered form of EGFR to expose domain II and increase scaffold binding (domain I, blue; domain II, green; domain III, yellow; domain IV, gray; EGF ligand, pink; scaffold, red). (B) The reactivity to EGFR in the presence of EGF (pink) or the absence of EGF (black). (C) The reactivity to EGFR fragment containing domains I–IV in the presence of EGF (orange) or the absence of EGF (black) as assessed by phage-ELISA. Error bars represent the standard deviation of phage-ELISA experiments performed with eight-fold replication.

We evaluated whether the scaffold binders showed higher reactivity to EGFR in the presence of the ligand EGF, which stabilizes EGFR in the tethered form to expose domain II (Figure 6A), using phage-ELISA with or without the addition of EGF. All six clones showed higher reactivity in the presence of EGF (pink bars) than in its absence (black bars; Figure 6B). This increased reactivity was not observed for wild-type 1OZJ, wild-type 1RK9, or the irrelevant control phage. To confirm these results, we performed phage-ELISA with eight-fold replication (i.e., eight samples for each phage clone) and found that the increased reactivity of the clones with the EGF treatment was significant (P value = 1.5×10^−4^ by a Wilcoxon rank-sum test; Table S1 in File S1).

We also evaluated whether the 1OZJ and 1RK9 clones showed a similar increase in reactivity to the extracellular domain of EGFR in the presence of EGF (Figure 6C). These results were similar to those obtained with whole EGFR: clones showed a higher affinity to EGFR domain I–IV in the presence of EGF (orange bars) than in its absence (black bars; Table S1 in File S1).

Interestingly, EGF treatment decreased the reactivity of cetuximab (i.e., a monoclonal antibody that targets EGFR) against both the whole EGFR protein (P value = 1.5×10^−4^; Figure 6B) and the EGFR extracellular domain (P value = 0.01; Figure 6C; Table S1 in File S1). This result is consistent with a previous observation that cetuximab binds to EGFR domain III, which is occluded by the binding of EGF [27].

## Discussion

In this study, we demonstrated a process to create protein interactions between EGFR domain II and computationally designed protein scaffolds. The two scaffolds, 1OZJ and 1RK9, previously had no common features except shape complementarity and favorable interaction energies with the target structure, EGFR domain II. By mutating several *in silico*-selected residues that had contributed to the unfavorable binding energies, we were able to produce novel protein–protein interactions for both 1OZJ and 1RK9 with EGFR domain II. We showed that the selected scaffolds specifically bound to EGFR domain II by showing that they did not bind to chimeric EGFR in which domain II was replaced by domain II of ErbB2 or ErbB4. We also confirmed that the designed scaffolds had a higher affinity to whole EGFR protein and to the extracellular domain of EGFR in the presence of the ligand EGF, which stabilizes EGFR in the untethered conformation to expose domain II [25], [27]. Our results demonstrate how non-interface protein surfaces can be changed to protein interfaces in a process that is independent from gene duplication.

Interestingly, the replaced residues of the high-reactivity binding scaffolds did not share properties of charge or hydrophobicity (Figure 3B). However, in both scaffolds, large amino acids in the binding interface were replaced with smaller amino acids. This result indicates that, in a protein pair with overall shape complementarity and favorable residue–residue interactions, the creation of a *de novo* interface may involve the elimination of steric hindrance on the surface patch. In nature, this change is likely to occur through accumulated point mutations.

This study has implications for the production of docking decoys. Protein docking simulation between two arbitrary proteins can generate hundreds or thousands of docking models. Non-native docking structures are usually considered artifacts. However, these structures may be considered candidates for *de novo* protein–protein interactions if these structures can be optimized to improve binding affinity.

We believe that computationally designed scaffolds have advantages over antibodies and conventional scaffolds. For example, our strategy enables a predetermined surface patch to be targeted at the start of the screening process. In contrast, the binding patch of conventional antibodies and single framework-dependent scaffolds is randomly determined. In addition, the process introduced in this paper selects epitope-specific frameworks. The structures that can be generated from this method are therefore more diverse than antibodies and single-framework-dependent scaffolds, because diversity in antibodies and single-framework-dependent scaffolds is inherently limited to their original template.

One limitation of our system is the need to produce a high-resolution structure of the target protein during the screening of the scaffold structure. Our method therefore cannot be used for targets with unknown structures. However, the scaffolds that can be generated using our computational method would make useful probes to screen for antibodies that bind to an epitope of interest, particularly because current epitope mapping methods are limited, and the identification of an epitope is not always easy. Current techniques, such as western-blot analysis, site-directed mutagenesis, and tandem mass spectroscopy, all have significant limitations. The antibody–antigen binding mode can be directly visualized at the atomic level by X-ray crystallography, which is probably the most definitive approach for epitope mapping [27], [36], [37]. X-ray crystallography is costly and time-consuming [38], however, and it is not always possible to crystallize the protein complex. Our computationally designed scaffolds can be used to screen for epitope-specific antibodies for use in competitive binding assays.

In summary, we used computational design and directed evolution to induce protein interactions between two previously unrelated proteins. Our results suggest that this method could be successfully applied to develop epitope-specific binder proteins for experimental or therapeutic use.

## Materials and Methods

### Construction of the human protein scaffold library

To construct a library that covers virtually all known human protein folds, we selected at most three representative protein folds from each structural class in the Structural Classification of Proteins database [39]. In structural classes consisting of fewer than three protein folds, we selected every available protein fold. However, only soluble and non-antibody proteins (molecular weight 10–40 kDa) were selected because low-molecular-weight proteins are likely to be overexpressed [7]. We eliminated membrane protein structures using annotations from the Protein Data Bank of Transmembrane Proteins [40], [41]. We eliminated antibodies using a keyword search in the Protein Data Bank [42]. To avoid promiscuous binding, we excluded proteins with >10 known interactions in the annotated Human Protein Reference Database [43]. We also excluded proteins that form homomultimers (other than homodimers and homotrimers) based on the SUBUNIT section of the Swiss-Prot database [44]. All the databases used in this study were downloaded in March of 2007.

### Screening scaffolds with shapes complementary to EGFR domain II

To screen the scaffolds for shapes complementary to EGFR domain II, docking simulations between each scaffold in the library and the A chain of the human EGFR extracellular domain (RCSB PDB ID: 1IVO) [26] were conducted using PatchDock (version 1.3) [31], [32]. For all other parameters in PatchDock, default values were used. The top 10 docking models were selected from each scaffold–EGFR docking result, and their binding modes were analyzed to find docking structures in which EGFR domain II was involved in the complex formation. A scaffold was considered to contact EGFR domain II in the docking complex if more than 10 of the following 28 residues of domain II were involved in the complex formation: 229, 230, 239, 242, 244, 245, 246, 248, 249, 250, 251, 252, 253, 262, 263, 264, 265, 275, 278, 279, 280, 282, 283, 284, 285, 286, 303, and 304. This residue numbering is consistent with the RCSB PDB structure of EGFR (1IVO). A residue was considered to be involved in binding if the residue underwent a change in solvent-accessible surface area >1 Å^2^ upon the formation of the docking complex between the two chains [45], [46]. The solvent-accessible surface area of each residue was calculated using NACCESS [47].

### Energy calculations for complex formation

The complex-formation energy and the energy contribution of each contact residue of the scaffolds were calculated using the EGAD program (April 2005 version) [35]. Using a docking structure generated by PatchDock as the template, JOBTYPE complex_formation_energy was used to calculate the binding energy; for all other options, default values were used. From the calculation result, the pseudo_DELTA_G_complex_formation value was used to evaluate complex formation energy, and dG values of level_1 interface residues were collected to determine unfavorable residues. Level_2 residues indirectly involved in the complex formation were excluded.

### Random library construction

The genes encoding the EGFR-binding scaffolds 1OZJ and 1RK9 were chemically synthesized (Genscript, Piscataway, NJ). Using these DNA molecules as templates, the target residues were randomized using NNK primers (Genotech Corporation, Daejeon, Korea). The following sequences of primers were used to randomize the five specific residues of the 1OZJ scaffold: primer 1: 5′ GG GCC CAG GCG GCC ATG TCG TCC 3′; primer 2: 5′ CTT GAG TTT CTT GAC CAG GCT CTT GAC CGC CTT CTC GCA 3′; primer 3: 5′ AGC CTG GTC AAG AAA CTC AAG NNK ACG GGG CAG CTG GAC GAG 3′; primer 4: 5′ GGG ATT CAC GCA GAC CTC MNN CTT CTT CAT MNN GAA GGC GAA MNN ACA CAG MNN CAT GGC CCG TAG CTC GTG 3′; and primer 5: 5′ GGC CGG CCT GGC CTG TGT GGC GTG GCA CCAACA CAG GAG GTA GAA CTG GTG TCT CTA CTC TCT GGT AGG GAT TCA CGC AGA CCT 3′. The following sequences of primers were used to randomize the six specific residues of 1RK9: primer 1: 5′ G GCC CAG GCG GCC ATG CGA ATG ACA GAC TTG CTG NNK GCT NNK GAC 3′; primer 2: 5′ CAG GCC GAC CAT TTG GAA GAA MNN MNN GTG GTC GAA GG 3′; primer 3: 5′ CC TTC GAC CAC NNK NNK TTC TTC CAA ATG GTC GGC CTG 3′; and primer 4: 5′ GGCC GGC CTG GCC TTA MNN TTC AGC CAC CAG MNN GG 3′.

The first polymerase chain reaction (PCR) amplification was performed in a 50-µl reaction volume containing 10 pmol of primers 1 and 2 or primers 3 and 4, 100 ng of the template DNA, 4 µl of 2.5 nM dNTP mix (Promega, Madison, WI), 10× reaction buffer, and 0.5 µl high-fidelity Taq polymerase (Roche, Indianapolis, IN). The PCR protocol consisted of 25 cycles of denaturation at 94°C for 15 s, annealing at 56°C for 30 s, and extension at 72°C for 90 s. Following 2% agarose gel electrophoresis, the specific DNA band was excised and purified using the QIAEX II Gel Extraction Kit (Qiagen).

To link the DNA fragment that was amplified with primers 1 and 2 with the DNA fragment that was amplified with primers 3 and 4, a second PCR amplification was performed in a 50-µl reaction volume containing 100 ng each of the two PCR products, 10 pmol of primers 1 and 4, 4 µl of 2.5 nM dNTP mix (Promega), 10× reaction buffer, and 0.5 µl high-fidelity Taq polymerase (Roche) using the same PCR protocol as described above. Linked DNA was confirmed and purified as described above. For the 1OZJ scaffold, one more step of PCR was performed with 10 pmol of primers 1 and 5 and the second PCR product as the template, using the same PCR protocol.

The PCR resulted in genes encoding the 1OZJ and 1RK9 scaffolds randomized at the selected residues and containing *Sfi* I restriction sites at the 5′ and 3′ ends. The scaffold DNA and phagemid vector pComb3X (Barbas III, 2001) were digested with *Sfi*I (Roche) and purified using the QIAEX II Gel Extraction Kit (Qiagen). The scaffold DNA and vector were ligated at 16°C overnight using T4 DNA ligase (Roche). The phage display library was constructed as previously described (Barbas III, 2001) the next day.

### Biopanning

Dynabeads (M-270 Epoxy, Invitrogen, Carlsbad, CA) were washed twice with 100 µl of sodium phosphate (0.1 M), and 5×10^7^ beads were conjugated to 15 µg of EGFR (Sigma, St. Louis, MO) according to the manufacturer's instructions. On the following day, the beads were washed four times with phosphate buffered saline (PBS; 137 mM sodium chloride, 10 mM phosphate, 2.7 mM potassium chloride, pH 7.4), incubated with 3% BSA in PBS for 1 h, and washed with 0.5% Tween-20 in PBS (PBST).

To enrich for specific EGFR binders, five rounds of biopanning were performed as described previously (Barbas III, 2001) with some modifications. Briefly, 10^7^ beads were used for the first round of biopanning, and 5×10^6^ beads were used for consecutive rounds of biopanning. EGFR-coated beads were incubated with 500 µl of randomized phage library for 2 h at room temperature in the rotator. A total of 10^12^ to 10^13^ phages were subjected to the first round of biopanning. Following an incubation period, the beads were washed with 0.05% TBST (v/v) to remove unbound phage one time during the first round of biopanning, three times during the second and third rounds of biopanning, and five times during the final two rounds of biopanning. Phages bound to the beads were eluted by adding 30 µl of glycine-HCl (0.1 M, pH 2.2) twice with 5-min incubations and neutralized with 2 M Tris-HCl (pH 9.1). Freshly grown *Escherichia coli* ER 2738 cells were infected with the eluted phages, and the phages were rescued by adding 10^12^ VCSM13 helper phage. Following an overnight culture, the rescued phages were precipitated from the culture supernatant for use in the next round of biopanning using polyethyleneglycol. During each round of biopanning, the numbers of input and output phages were titrated.

### Expression and purification of EGFR fragments

EGFR cDNA was obtained from the Korea Human Gene Bank (Daejeon, Korea). Primers were synthesized (Genotech) to produce DNA fragments of EGFR domains I–II and I–IV with *Sfi* I restriction enzyme sites at the 5′ and 3′ ends. The following primer sequences were used for EGFR domain I–II: (forward) 5′ GGC CCA GGC GGC CCT GGA GGA AAA GAA AGT TTGC 3′ and (reverse) 5′ GGC CGG CCT GGC C GCG GCA AGG CCC TTC GC 3′. The following primer sequences were used for EGFR domain I–IV: (forward) 5′ GGC CCA GGC GGC CCT GGA GGA AAA GAA AGT TTGC 3′ and (reverse) 5′ GGC CGG CCT GGC C GCG GCA AGG CCC TTC GC 3′.

The EGFR fragments were amplified by PCR in a 50-µl reaction volume containing 100 ng of EGFR cDNA, 10 pmol of sense and reverse primers (specific to either domain I–II or I–IV), 4 µl of 2.5 nM dNTP mix (Promega), 10× reaction buffer, and 0.5 µl high-fidelity Taq polymerase (Roche). The PCR protocol consisted of 20 cycles of denaturation at 94°C for 15 s, annealing at 56°C for 30 s, and extension at 72°C for 90 s. Following 1% agarose gel electrophoresis, the specific DNA band was excised and purified using the QIAEX II Gel Extraction Kit (Qiagen). A modified pCEP4 vector containing the human IgG1 Fc fragment (Park et al, 2010) and the purified PCR products of EGFR domain I–II and EGFR domain I–IV were simultaneously digested with *Hind*III and *BamH*I (New England Biolabs, Ipswich, MA) at 37°C overnight. The inserts were ligated into the pCEP4 vector and transformed into the *E. coli* strain DH5α (Invitrogen). The plasmid DNA was prepared using the MG™ Plasmid SV kit (Macrogen, Daejeon, Korea) for transfection into human embryonal kidney (HEK) 293F cells (Invitrogen).

Gene constructs encoding the chimeric EGFR domain I–IV fragments in which the EGFR domain II was replaced with domain II of ErbB2 and ErbB4, respectively, were synthesized (Genscript) with *Sfi* I restriction sites at the 5′ and 3′ ends. The constructs were digested and ligated with pCEP4 vector.

The HEK 293F cells were cultured in GIBCO FreeStyle 293 Expression medium (Invitrogen) in Erlenmeyer tissue culture flasks (Corning Inc., Corning, NY) at 135 rpm at 37°C with 8% CO_2_ on an orbital shaking incubator (Minitron, INFORS HT, Switzerland). Fresh medium was added the day before transfection to achieve a density of 1.0×10^6^ cells/ml, which resulted in a density of 2.0×10^6^ cells/ml on the day of transfection. HEK293F cells were transfected with the expression vectors using Lipofectamine™ 2000 (Invitrogen) according to the manufacturer's instructions. The transfected cells were cultured for 3 more days, and then the culture supernatants were harvested. Following filtration through a membrane (Advantec, Toyo Roshi Kaisha, Ltd., Japan), the culture supernatant was subjected to gel affinity chromatography (IPA 300 Protein A Affinity Resin, RepliGen, Waltham, MA). The purified EGFR fragments were analyzed by SDS-PAGE using NuPAGE 4%–12% Bis-Tris gel (Invitrogen).

### Phage-ELISA

Phage-ELISA was performed as described previously (Barbas III, 2001) with some modifications. Individual colonies were randomly selected from the output titration plate on the last round of biopanning and inoculated into 1 mL of super broth (3% tryptone, 2% yeast extract, 1% 3-[N-Morpholino] propanesulfonic acid, pH 7.0). The phages were rescued by adding 10^12^ VCSM13 helper phage. Following overnight growth at 37°C, the culture supernatant was analyzed by ELISA. A 96-well microtiter plate was coated overnight in 20 µl of PBS at 4°C with 4 µg/mL of EGFR (Sigma), the EGFR domain I–II fragment, the EGFR domain I–IV fragment, or the chimeric EGFR domain I–IV fragment with EGFR domain II replaced by ErbB2 domain II and ErbB4 domain II, respectively. The plate was washed twice with PBST and incubated with 150 µl of BSA (3%) in PBS for 1 h at 37°C. The phage-containing culture supernatant (25 µl) was mixed with 25 µl of BSA (6%) in PBS and added to each well. Following a 1-h incubation at 37°C, the plate was washed three times with PBST. Horseradish peroxidase-conjugated anti-M13 antibody (50 µl; Sigma) diluted with 3% BSA in PBS (1∶5,000) was added to the plate, which was then incubated for 1 h at 37°C and washed with 0.05% PBST. For cetuximab, horseradish peroxidase-conjugated anti-Human Fc antibody (Sigma) was used. A substrate solution (3,3′,5,5′-tetramethylbenzidine; 50 µl; Pierce, Rockford, IL) was added to each well, and the optical density (OD) was measured at 650 nm. Phage titers in the culture supernatant were determined as described previously (Barbas III, 2001) to compare the reactivity of positive phage clones, and the same number of phages was used. All phage-ELISA experiments were performed in triplicate.

### ELISA with the addition of EGF

Selected phage clones expressing EGFR binders were amplified as described above. A 96-well microtiter plate was coated with 4 µg/ml of EGFR (Sigma) or EGFR domain I–IV in 20 µl of PBS at 4°C overnight. Following a 1-hr incubation with 150 µl of BSA (3%) in PBS at 37°C, a mixture of 25 µl of phage supernatant and 25 µl of EGF (160 ng/ml) in 6% BSA in PBS was added, and the assay was conducted as described above. Eight wells were used for each phage clone for maximum clarity. P-values for each template were calculated using the Wilcoxon rank-sum test.

## Supporting Information

File S1 **Supporting Dataset and Table.** **Dataset S1.** Human scaffold library. Research Collaboratory for Structural Bioinformatics Protein Data Bank (PDB) accession numbers of 717 proteins in human scaffold library. The first four characters indicate the PDB accession number. For proteins with more than one chain, the chain we used was specified after the colon. **Table S1.** Statistical significance of the difference in binding affinity in the presence of exogenous EGF, as determined by the Wilcoxon rank-sum test.
(DOC)
