# Supplementary material for: Computational Design of Binding Proteins to EGFR Domain II
Source: PLoS One. 2014 Apr 7;9(4):e92513. doi: 10.1371/journal.pone.0092513 (PMC3977815; doi:10.1371/journal.pone.0092513)
Supplement: File S1 — Supporting Dataset and Table. Dataset S1. Human scaffold library. Research Collaboratory for Structural Bioinformatics Protein Data Bank (PDB) accession numbers of 717 proteins in human scaffold library. The first four characters indicate the PDB accession number. For proteins with more than one chain, the chain we used was specified after the colon. Table S1. Statistical significance of the difference in binding affinity in the presence of exogenous EGF, as determined by the Wilcoxon rank-sum test. (DOC) [file pone.0092513.s001.doc]

**Computational Design of Binding Proteins to EGFR Domain II**

Yoon Sup Choi, Soomin Yoon, Kyung-Lock Kim, Jiho Yoo, Parkyong Song, Minsoo Kim, Young-Eun Shin, Won Jun Yang, Jung-eun Noh, Hyun-soo Cho, Sanguk Kim, Junho Chung, and Sung Ho Ryu

**Dataset S1**.

| 10GS:A | 133L | 1A1Z | 1A31:A | 1A3S | 1A4I:A | 1A4I:B |
| --- | --- | --- | --- | --- | --- | --- |
| 1A4Y:D | 1A6Q | 1A9N:C | 1ABN | 1ANP | 1APZ:C | 1AUK |
| 1AUT:L | 1AV1:D | 1AV5:A | 1AVO:M | 1AWB:A | 1AYE:1 | 1AYE:4 |
| 1AYP:A | 1B1E:A | 1B22:A | 1B64 | 1BA9 | 1BEH:B | 1BF5:A |
| 1BHG:A | 1BIC | 1BIX | 1BKC:A | 1BMQ:A | 1BNK:A | 1BO1:A |
| 1BSI:1 | 1BSI:4 | 1BV8:A | 1BVK:A | 1BX4:A | 1BY2 | 1BYW:A |
| 1C4Z:A | 1CA7:A | 1CB3:A | 1CDL:A | 1CDZ:A | 1CF7:B | 1CJY:B |
| 1CQR:A | 1CW3:A | 1CZA:N | 1D1S:A | 1D3B:L | 1D3H:A | 1D3L:A |
| 1D6G:A | 1D7K:B | 1D8K:A | 1DAN:L | 1DAN:T | 1DE4:I | 1DEB:B |
| 1DEE:H | 1DFC:B | 1DHS | 1DKT:B | 1DMW:A | 1DNC:1 | 1DNC:3 |
| 1DNG:A | 1DO8:A | 1DPU:A | 1DRZ:A | 1DT7:Y | 1DT9:A | 1DTD:B |
| 1DTW:A | 1DTW:B | 1DY0:A | 1DYK:A | 1E2D:A | 1E41:A | 1E7K:B |
| 1E8S:B | 1EBM:A | 1EBP:D | 1EES:B | 1EF1:D | 1EFN:D | 1EGX:A |
| 1EI0:A | 1EJF:B | 1EJQ:B | 1EM6:A | 1EQX:A | 1EW2:A | 1EWF:A |
| 1EYB:A | 1EZF:A | 1F3M:B | 1F3U:H | 1F3V:A | 1F59:D | 1F5N:A |
| 1F95:B | 1FAK:I | 1FB1:A | 1FBV:A | 1FH0:A | 1FJ2:A | 1FKN:A |
| 1FKR | 1FO3:A | 1FS2:C | 1FWQ:A | 1FXT:B | 1FYJ:A | 1G0Y:I |
| 1G2S:A | 1G55:A | 1G73:B | 1GJJ:A | 1GO4:H | 1GQB:B | 1GVL:A |
| 1GW3 | 1GW5:B | 1GW5:S | 1GW6:A | 1GZS:D | 1H0Z:A | 1H4O:A |
| 1H4U:A | 1H6E:A | 1H6P:B | 1H7C:A | 1H7U:B | 1HD4:A | 1HE1:B |
| 1HE8:A | 1HE8:B | 1HLG:B | 1HLV:A | 1HNN:A | 1HOF:A | 1HRK:A |
| 1HTI:A | 1HWL:D | 1HY9:A | 1HYN:S | 1HZD:F | 1HZF:A | 1I16 |
| 1I2M:D | 1I4T:B | 1I72:B | 1IAP:A | 1IAT:A | 1IB1:A | 1IB1:E |
| 1IBX:B | 1IH5:A | 1IIE:C | 1IK9:B | 1IKT:A | 1IL0:A | 1IM3:P |
| 1IMJ:A | 1INZ:A | 1IO4:D | 1IPC:A | 1IQ3:A | 1IR3:A | 1IRY:A |
| 1ISM:B | 1ITU:B | 1IUR:A | 1IV5:B | 1IVO:b | 1IVT:A | 1IXD:A |
| 1J04:A | 1J1E:E | 1J1E:F | 1J1J:D | 1J1L:A | 1J3S:A | 1JB6:B |
| 1JBI:A | 1JBQ:A | 1JDP:B | 1JDW | 1JEY:A | 1JEY:B | 1JH4:A |
| 1JJQ:A | 1JKY:A | 1JLJ:A | 1JMT:A | 1JN5:B | 1JOC:B | 1JPW:F |
| 1JPY:Y | 1JQE:B | 1JR2:B | 1JSU:C | 1JU6:A | 1JV1:A | 1JWH:C |
| 1JXS:A | 1K1F:H | 1K5G:L | 1K62:A | 1K6M:B | 1K8F:D | 1K8O:A |
| 1KBH:B | 1KBO:A | 1KCW:8 | 1KEX:A | 1KFX:L | 1KFX:S | 1KJ6:A |
| 1KJS | 1KJY:C | 1KKU:A | 1KN0:K | 1KO6:C | 1KOO:C | 1KOO:D |
| 1KOY:A | 1KPS:B | 1KR5:A | 1KSW:A | 1KU6:A | 1KU6:B | 1KY7:A |
| 1KZY:D | 1L2Z:A | 1L3E:A | 1L3E:B | 1L4T:A | 1L4Z:B | 1L6L:Z |
| 1L9M:A | 1L9X:D | 1LCY:A | 1LI1:F | 1LI4:A | 1LIY:D | 1LJ2:B |
| 1LJ7:J | 1LJM:B | 1LL8:A | 1LM7:B | 1LN3:B | 1LP3:A | 1LQF:A |
| 1LS6:A | 1LSL:A | 1LT8:B | 1LV4:A | 1LVR:A | 1LY7:A | 1M12:A |
| 1M1L:D | 1M36:A | 1M4F:A | 1M4U:A | 1M5I:A | 1M7K:A | 1M7R:B |
| 1M8Z:A | 1MA3:A | 1MEN:A | 1MFQ:B | 1MHL:A | 1MJ4:A | 1MJE:A |
| 1MK2:B | 1MKC:A | 1MKN:A | 1ML0:A | 1MM3:A | 1MP1:A | 1MQ0:A |
| 1MR0:A | 1MR1:D | 1MWP:A | 1MX3:A | 1MXL:I | 1MZF:A | 1N0W:B |
| 1N3U:A | 1N5G:A | 1N69:C | 1N72:A | 1N7D:A | 1N8S:A | 1N8S:C |
| 1N9V:A | 1NB8:A | 1NE7:A | 1NF7:A | 1NHX:A | 1NN8:T | 1NNL:B |
| 1NRG:A | 1NU9:F | 1NUB:B | 1NVP:D | 1NW3:A | 1NWU:D | 1O4X:B |
| 1O5T:A | 1O6S:A | 1O70:A | 1O77:E | 1O7A:A | 1O7K:C | 1O86:A |
| 1O8R:A | 1O9K:H | 1OA8:D | 1OAI:A | 1OAT:A | 1OE9:A | 1OEI:A |
| 1OG2:A | 1OKI:B | 1OLM:E | 1ONI:I | 1ONV:A | 1OQJ:B | 1OQX:D |
| 1OR3:A | 1ORE:A | 1OTH:A | 1OU5:A | 1OV2:A | 1OW1:A | 1OW8:C |
| 1OWT:A | 1OZJ:B | 1P22:A | 1P22:B | 1P27:C | 1P32:C | 1P3P:H |
| 1P4R:A | 1P4U:A | 1P57:A | 1P6A:A | 1P97:A | 1P9O:B | 1PB5:A |
| 1PBU:A | 1PCF:H | 1PFJ:A | 1PFL | 1PI1:A | 1PIN:A | 1PJA:A |
| 1PLP | 1POU | 1PQ3:F | 1PS2 | 1PT9:B | 1PV8:B | 1PWB:C |
| 1PY1:A | 1Q0B:B | 1Q1V:A | 1Q2H:C | 1Q3L:A | 1Q3X:A | 1Q5H:A |
| 1Q5W:A | 1Q69:A | 1Q69:B | 1Q7D:C | 1Q7L:A | 1Q7S:B | 1Q8K:A |
| 1Q92:A | 1Q9C:I | 1Q9P:A | 1Q9S:A | 1QB0:A | 1QB2:B | 1QBJ:A |
| 1QCM | 1QGT:D | 1QGV:A | 1QH5:B | 1QIN:A | 1QK1:H | 1QK9:A |
| 1QKI:A | 1QKL:A | 1QM3:A | 1QO5:A | 1QQW:D | 1QRJ:A | 1QRJ:B |
| 1QTU:A | 1QU6:A | 1QWT:B | 1QZU:D | 1R0D:I | 1R21:A | 1R3Q:A |
| 1R4M:A | 1R4X:A | 1R55:A | 1R5H:A | 1R5I:H | 1R5L:A | 1R74:B |
| 1RF1:F | 1RF3:A | 1RF3:B | 1RGO:A | 1RH0:B | 1RHO:C | 1RI0:A |
| 1RK9:A | 1RKP:A | 1RMJ:A | 1RN7:A | 1RRJ:A | 1RRP:D | 1RW2:A |
| 1RX0:A | 1RY1:W | 1RYU:A | 1RZ4:A | 1RZK:G | 1S1C:Y | 1S1D:B |
| 1S1G:B | 1S1Q:C | 1S2H:A | 1S35:A | 1S3E:B | 1S3U:A | 1S4J:A |
| 1S4X:A | 1S4Y:C | 1S5O:A | 1S5Q:A | 1S5Q:B | 1S78:B | 1S7A:A |
| 1S9D:E | 1SBX:A | 1SGH:A | 1SGH:B | 1SGO:A | 1SHW:A | 1SHW:B |
| 1SHY:B | 1SI3:A | 1SK6:C | 1SKO:B | 1SMB:A | 1SO0:D | 1SRA |
| 1SRQ:D | 1SS6:A | 1SSU:A | 1ST0:A | 1ST0:B | 1SU3:B | 1SUV:F |
| 1SVC:P | 1SX6:A | 1SXE:A | 1SYQ:A | 1SZB:B | 1T08:B | 1T0J:C |
| 1T0L:D | 1T2F:D | 1T2K:B | 1T2K:D | 1T2V:E | 1T39:B | 1T5Q:A |
| 1T5Y:A | 1T6B:X | 1T77:D | 1T7H:B | 1T84:A | 1T8P:B | 1T94:B |
| 1T9G:S | 1TA0:A | 1TDH:A | 1TE6:B | 1TF0:B | 1TFF:A | 1TJJ:C |
| 1TKN:A | 1TNR:R | 1TNZ:K | 1TNZ:L | 1TP4:A | 1TPN | 1TQE:S |
| 1TWQ:A | 1TXU:A | 1TZN:O | 1U32:A | 1U5M:A | 1U6D:X | 1U6G:A |
| 1U6G:C | 1U7B:A | 1U8C:B | 1UAD:D | 1UCF:B | 1UCH | 1UCN:C |
| 1UCP:A | 1UCV:A | 1UEL:B | 1UF0:A | 1UFI:D | 1UGH:E | 1UGH:I |
| 1UII:B | 1UJ2:B | 1UJS:A | 1UK1:B | 1UKL:B | 1UKL:F | 1UMK:A |
| 1UMW:B | 1UOL:A | 1UOR:4 | 1UOU:A | 1UPL:B | 1UPT:H | 1URF:A |
| 1US7:B | 1USE:A | 1UTC:B | 1UUH:B | 1UW0:A | 1UW2:A | 1UW4:A |
| 1UW4:B | 1UW5:D | 1UWY:A | 1UX6:A | 1UZC:A | 1UZQ:A | 1V04:A |
| 1V05:A | 1V45:E | 1V49:A | 1V5W:B | 1V82:A | 1V95:A | 1V9U:4 |
| 1VAR:B | 1VCB:K | 1VCB:L | 1VCU:B | 1VD4:A | 1VF6:D | 1VFE |
| 1VHR:B | 1VJ5:A | 1VOL:A | 1VOL:B | 1VYW:D | 1W0U:B | 1W1I:H |
| 1W22:B | 1W2F:B | 1W3B:B | 1W4M:A | 1W6K:A | 1W7L:A | 1W9C:B |
| 1WD8:A | 1WF6:A | 1WGL:A | 1WGM:A | 1WGO:A | 1WGV:A | 1WGX:A |
| 1WH0:A | 1WH9:A | 1WHB:A | 1WHR:A | 1WI3:A | 1WI5:A | 1WIG:A |
| 1WIL:A | 1WIZ:A | 1WJ4:A | 1WJI:A | 1WJO:A | 1WJS:A | 1WLJ:A |
| 1WMH:B | 1WNJ:A | 1WOU:A | 1WQ1:G | 1WUU:D | 1WWW:W | 1X79:A |
| 1X79:C | 1X8Y:A | 1X9N:A | 1XA6:A | 1XAR:B | 1XB1:F | 1XBT:H |
| 1XDT:T | 1XDV:B | 1XFD:D | 1XFE:A | 1XJL:B | 1XK8:F | 1XKI:A |
| 1XKS:A | 1XKT:B | 1XKU:A | 1XMJ:A | 1XNI:J | 1XNT:A | 1XPA |
| 1XPW:A | 1XQ8:A | 1XQH:E | 1XR0:B | 1XT9:A | 1XTG:A | 1XTG:B |
| 1XTK:A | 1XU1:A | 1XU1:R | 1XWE:A | 1XXF:D | 1XZ6:A | 1Y01:A |
| 1Y02:A | 1YCR:A | 1YET | 1YGS | 1YVN:A | 1YVN:G | 1ZOP:B |
| 1ZTO | 1ZWG | 2A5E | 2AW0 | 2BBY | 2BFH | 2BID:A |
| 2BJX:A | 2DYN:B | 2EZG:A | 2EZZ:B | 2FHA | 2FMR | 2FN2 |
| 2GLI:A | 2HGF | 2HGS:A | 2HP8 | 2HPA:D | 2HPP:P | 2IF1 |
| 2ILK | 2NGR:B | 2NLL:B | 2NR1 | 2PRG:C | 2REL | 2SEB:D |
| 2STW:A | 2TMP | 2VGH | 2VPF:H | 3BMP:A | 3FIB | 3HHR:A |
| 3HLA:A | 3IFB:A | 3INK:D | 3PDZ:A | 3PSR:B | 3SAK:D | 3ULL:B |
| 3YGS:P | 4FAP:B | 4HTC:I | 4LBD | 4NOS:D | 4TRX | 5CYH:A |
| 5GAL:B | 5I1B | 5PNT | 5TTR:H | 6HBW:D | 6PAX:A | 6RLX:D |
| 8FAB:D | 9ICY:A | 9PAI:A |  |  |  |  |

**Table S1.** Statistical significance of the difference in binding affinity in the presence of exogenous EGF, as determined by the Wilcoxon rank-sum test.

|  | 1OZJ | | | | | 1RK9 | cetuximab |
| --- | --- | --- | --- | --- | --- | --- | --- |
|  | 6 | 9 | 2-2 | 2-16 | 2-17 | 2-31 |
| Whole EGFR protein | 1.5×10^-4^ | 1.5×10^-4^ | 1.5×10^-4^ | 1.5×10^-4^ | 1.5×10^-4^ | 1.5×10^-4^ | 1.5×10^-4^ |
| EGFR fragment containing domain I–IV | 0.01 | 1.5×10^-4^ | 1.5×10^-4^ | 1.5×10^-4^ | 1.5×10^-4^ | 1.5×10^-4^ | 0.01 |
